# Description of Hip Deformities in 5-Year-Old Patients with Congenital Zika Virus Syndrome

**DOI:** 10.1055/s-0045-1814120

**Published:** 2025-12-30

**Authors:** Amnuay Kleebayoon, Viroj Wiwanitkit

**Affiliations:** 1 Private Academic Consultant, Samraong, Cambodia; 2 Department of Community Medicine, D Y Patil Medical College, Hospital and Research Centre, Dr. D Y Patil Vidyapeeth (Deemed to be University), Pune, India

Dear Editor,

The purpose of the study “Description of Hip Deformities in 5-Year-Old Patients with Congenital Zika Virus Syndrome: A Cross-Sectional Study” 1 was to look at common hip anomalies in children over the age of five with congenital Zika virus syndrome (CZS) using clinical and radiographic examinations. Although the study provides helpful information for a specific patient population, it has methodological and statistical limitations that must be carefully evaluated. One example is the study's cross-sectional and retrospective design, which may add selection bias and limit the capacity to accurately discern cause and effect between variables.

The sample size of only 62 patients may be insufficient for complicated statistical analyses, particularly when stratified by GMFCS level, resulting in fewer individuals per group and lower precision and statistical power. Furthermore, no confidence intervals or controls for confounding variables, such as other medical histories or nutritional factors, that could influence hip structural development were given. Although comparison data were available, the relationships between variables such as Reimers Index (RI), acetabular index (AI), femoral neck-shaft angle (FNSA), and tenotomy did not clarify how the covariates were controlled or matched, which limited the results' interpretation.

Questions to inspire academic discussion include: Does the association between GMFCS and radiographic changes reflect genuine illness development, or is it merely the product of differences in movement habits between groups? Is the lack of a difference in FNSA across groups clinically significant? Is it related to sample size limitations? Another intriguing finding is that, while tenotomy increases leg extension, it has no effect on radiographic values, implying that surgical therapies should be evaluated for more than simply short-term clinical results.

Future studies should include cohort studies to evaluate long-term radiographic changes and mobility. Furthermore, sample sizes should be larger, and data from various locations should be incorporated to improve the generalizability of the findings. New technologies, such as 3D modeling of radiographic images or artificial intelligence for image analysis, should be employed to determine the intricate links between anatomical features and clinical outcomes, allowing for more effective individualized care in CZS patients.
